# A 24-Hour Temporal Profile of *In Vivo* Brain and Heart PET Imaging Reveals a Nocturnal Peak in Brain ^18^F-Fluorodeoxyglucose Uptake

**DOI:** 10.1371/journal.pone.0031792

**Published:** 2012-02-22

**Authors:** Daan R. van der Veen, Jinping Shao, Sarah Chapman, W. Matthew Leevy, Giles E. Duffield

**Affiliations:** 1 Department of Biological Sciences, Galvin Life Science Center, University of Notre Dame, Notre Dame, Indiana, United States of America; 2 Department of Physiology, Nankai University School of Medicine, Tianjin, People's Republic of China; 3 Notre Dame Integrated Imaging Facility, University of Notre Dame, Notre Dame, Indiana, United States of America; 4 Department of Chemistry and Biochemistry, University of Notre Dame, Notre Dame, Indiana, United States of America; Vanderbilt University, United States of America

## Abstract

Using positron emission tomography, we measured *in vivo* uptake of ^18^F-fluorodeoxyglucose (FDG) in the brain and heart of C57Bl/6 mice at intervals across a 24-hour light-dark cycle. Our data describe a significant, high amplitude rhythm in FDG uptake throughout the whole brain, peaking at the mid-dark phase of the light-dark cycle, which is the active phase for nocturnal mice. Under these conditions, heart FDG uptake did not vary with time of day, but did show biological variation throughout the 24-hour period for measurements within the same mice. FDG uptake was scanned at different times of day within an individual mouse, and also compared to different times of day between individuals, showing both biological and technical reproducibility of the 24-hour pattern in FDG uptake. Regional analysis of brain FDG uptake revealed especially high amplitude rhythms in the olfactory bulb and cortex, while low amplitude rhythms were observed in the amygdala, brain stem and hypothalamus. Low amplitude 24-hour rhythms in regional FDG uptake may be due to multiple rhythms with different phases in a single brain structure, quenching some of the amplitude. Our data show that the whole brain exhibits significant, high amplitude daily variation in glucose uptake in living mice. Reports applying the 2-deoxy-D[^14^C]-glucose method for the quantitative determination of the rates of local cerebral glucose utilization indicate only a small number of brain regions exhibiting a day versus night variation in glucose utilization. In contrast, our data show 24-hour patterns in glucose uptake in most of the brain regions examined, including several regions that do not show a difference in glucose utilization. Our data also emphasizes a methodological requirement of controlling for the time of day of scanning FDG uptake in the brain in both clinical and pre-clinical settings, and suggests waveform normalization of FDG measurements at different times of the day.

## Introduction

Positron Emission Tomography (PET) is a non-invasive and quantitative nuclear imaging modality used for a range of clinical diagnostic and pre-clinical experimental applications. PET often employs ^18^F-fluorodeoxyglucose (FDG), a sugar molecule labeled with the positron emitting radionucleotide ^18^F (110 minute half-life), as the nuclear probe for imaging [1]. FDG is taken up by cells and subsequently phosphorylated, but cannot be metabolized any further [2], thus providing a measure of glucose uptake by cells. This imaging technique provides functional information in detecting tissues with high glucose demands such as the heart, the brain, and many types of cancers. Scanning of the radiolabeled glucose in a three dimensional space in living tissue is a powerful tool in imaging the metabolic condition of a tissue of interest. PET scanning is used in a wide array of pre-clinical and clinical settings to investigate of diseases such as dementia, Alzheimer's disease, Parkinson's disease, brain injury, stroke, coronary heart disease and oncology [3]–[5]. As a matter of practicality, FDG is synthesized during the early morning hours for usage by clinicians and researchers during the daytime. Because of the short half-life of the radionucleotide this introduces a peculiar dichotomy in which human patients are imaged during their “awake” phase of the day, while pre-clinical specimens like nocturnal mice are imaged during their “sleep” phase, unless they are kept under light-dark conditions that are different from the normal day timing.

Many processes in physiology, vigilance state and cognitive performance are widely observed to show daily (or ‘diel’) variation. This daily pattern can be driven by homeostatic processes in physiology (e.g. tiredness, hunger) and/or by cues from the external environment (e.g. changes in activity pattern due to environmental light-dark conditions or social obligations). When the daily rhythms in variation persist under constant conditions, they are said to be ‘circadian’ (∼24 hour), and these circadian rhythms are driven by internal clocks. A biological clock, located in the brain, is synchronized (entrained) by the environmental light-dark (LD) cycle, and coordinates the anticipation of many of these daily, rhythmic processes through transmitting timing information to a hierarchy of clocks in the central nervous system (CNS) and peripheral clocks [6]. This principal light entrainable clock in mammals is located in the suprachiasmatic nuclei (SCN) of the hypothalamus and drives rhythms in behavior and physiology [7].

Quantifying temporal glucose *utilization* of brain regions has been made possible in terminal experiments using the 2-deoxy-D[^14^C]-glucose (2-DG) method originally described by Sokoloff *et al.* [8]. Diurnal rhythmicity in glucose utilization in the SCN has been reported to be high during the day across mammalian species, including night active, day active and bimodal/crepuscular animals (e.g. mouse [9], opossum [10], rat, monkey and cat [11]). Also in birds, the visual SCN shows rhythmic 2-DG uptake, peaking during the subjective day (House sparrow [12] and chicken [13]) under both LD and constant darkness (DD) conditions. The rhythm in SCN glucose metabolism is related to SCN function (see [14]) and is in antiphase with rhythms of glucose uptake or utilization observed in other brain regions.

In addition to the SCN, many structures in the CNS have been found to show daily profiles in expression of one or more clock genes ([15] and referenced therein for central, extra-SCN oscillators). While cells and tissues can show clock gene expression with daily patterning, this does not mean that they are self-sustained oscillators, and expression can be driven by rhythmic processes. Using the 2-DG method throughout the brain, Crane *et al*. [16] reported a 24-hour variation in cortical glucose utilization in rats with a higher utilization during dark than light, showing peak values during the night phase (Zeitgeber Time [ZT] 15; ZT 0 is lights on and ZT 12 is lights off). When looking at the quantitation of glucose utilization in different brain structures of female mice using the 2-DG method, 8 out of 60 structures identified showed significantly lower glucose utilization between ZT 6–8 compared to ZT 18–20 [9]. Similarly in freely moving rats, glucose utilization as quantified using the 2-DG method revealed a day-night variation with increased glucose utilization in 17 out of 144 brain structures in the first 4 hours of the dark phase of the LD cycle [17]. In both studies, only one brain structure, the SCN, showed significantly higher levels of glucose utilization during the daytime phase [9], [17]. Karaganis *et al*. [18] showed that the diurnal leghorn chicken shows peaks in 2-DG uptake in the telen- and diencephalon during mid, to late day (peaking at ∼ZT 8) under LD cycle conditions, but this was not observed in DD. These authors also showed a rhythm in heart and liver 2-DG uptake, with peaks in the dark phase, liver peaking around ZT 17 and heart around ZT 15. Peripheral rhythms in 2-DG uptake did not persist in DD, when rhythmicity is self-driven, as opposed to driven by the external LD cycle.

Energy utilization of central tissues is to a large extent attributed to action potentials and postsynaptic potentials [19]. Energy demands of the brain account for up 20% of the standard metabolic rate of the whole body [20] and thus it utilizes a large proportion of the circulating blood glucose. The daily rhythm in activity and inactivity of numerous processes in physiology and behavior driven by the CNS leads to the hypothesis that the brain utilizes glucose in a pattern that shows a high daily amplitude. The autoradiographic 2-deoxy-D[^14^C]-glucose method of Sokoloff *et al.* [8] is very effective in the quantitative determination of the rates of local cerebral glucose utilization, but does not present a uniform, within individual pattern of underlying brain glucose uptake, and where uptake reflects the energy demand that effectively needs to be met by the cells.

The heart is among the higher oxygen consuming tissues [20] and is visible on PET scans as a major organ of FDG uptake. A temporal profile with an early morning peak in acute coronary heart disease and cardiac death is observed [21] and expression of the molecular components of the clock is apparent in cardiomyocytes and persists independently in an *in vitro* culture [22], [23], indicating a functional clock mechanism. Indeed, Young and colleagues [24] found that rat hearts had higher carbohydrate oxidation, contractile function and output during the middle of the dark phase of the LD cycle as compared to the middle of the light phase. Moreover, in the same study in rats, gene expression for the GLUT1 and GLUT4 glucose transporters was up regulated in the early dark phase, which is the active phase for these nocturnal animals.

Previous measurements using the 2-DG method only report on site-specific (as opposed to whole individual) glucose utilization and not uptake from the glucose stores in the circulating blood in real-time. Additionally, quantifications of glucose utilization were made at one time point only for each individual animal. Using PET, we can measure glucose uptake in whole brain and heart in living mice throughout the 24-hour LD cycle, including multiple measurements in a single animal. PET is increasingly applied in rodent models and in clinical applications [5] and daily variation in brain glucose uptake may mask some of the results found.

Given that mouse models of disease are intended to mirror those taking place in humans, we sought to determine if the synchronization of FDG-PET imaging with circadian biology would result in signal variation from relevant tissues in mice. Thus, we have employed PET imaging on C57Bl/6 mice at multiple times of day within and between individuals to interrogate the effects of daily rhythms on FDG uptake in brain and heart tissue. Using this method we show strong time-of-day specific variation in glucose uptake at the level of the whole brain and variant amplitudes in different brain regions. Additionally, we show that heart glucose uptake in the same animals does not show such rhythmicity, but shows high variability throughout the 24-hour day. These data show a much larger scale of 24-hour variation in glucose demand than would be expected from previously reported glucose utilization studies using the 2-DG method. It also indicates that for both clinical and pre-clinical/experimental applications of PET scanning, time-of-day of measurement is an important variable that must be considered.

## Results

**Figure 1:**
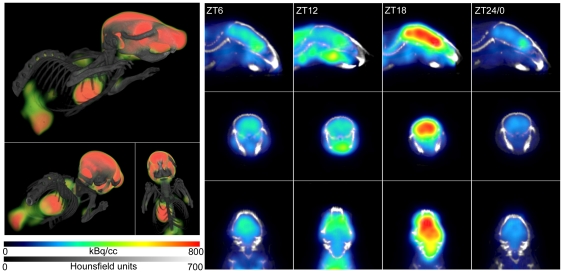
Fused CT and PET images from individual representative C57Bl/6 mice. The left panel shows a 3D reconstruction of a mouse measured in the middle of the dark phase of the light-dark cycle, where brain, heart and bladder show high FDG uptake. Both kidneys and the interscapular brown adipose tissue are also visible. Colors indicate only mid-range FDG uptake, where green is lower and red is higher uptake. On the right, representative individuals from the 4 groups measured at (from left to right) ZT6, 12, 18 and 24/0 are depicted. Images are shown for sagittal (top), coronal (middle) and transverse (bottom) planes. Scale bars belonging to the right panel for CT intensity (represented as gray-scale) and FDG uptake (in color) are shown on the bottom left.

The left frames of Figure 1 show a 3-dimensional reconstruction of merged X-ray computerized tomography (CT, greyscale) and PET (color scale) images of a mouse at the middle of the dark phase of the LD cycle. The PET shows the high localization of (^18^F) fluorodeoxyglucose (FDG) in the brain and heart. The right panels in Figure 1 show representative images of individual mice at 4 times during the 24-hour LD cycle used for glucose uptake measurements. When multiple measurements were made within one mouse, they are included. Mean age at the time of measurement was 92 days (SEM = 5 days) and was not different between animals measured at different times of day (proc mixed P>0.05). Both for brain and heart tissue, there was no effect of sex of the animal on the total FDG uptake (proc mixed, P's>0.05). Mean volumes in the region-of-interest were 0.462 (SEM = 0.005) cubic centimeter (ccm) for the brain and 0.230 (SEM = 0.013) ccm for the heart, and these volumes did not differ between time-of-day or sexes (proc mixed P's>0.05).

**Figure 2:**
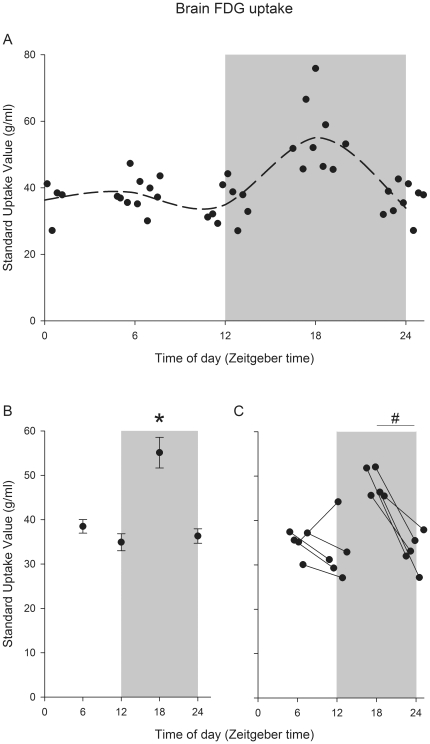
Time-of-day specific analysis of FDG uptake in brain. (A) Whole Brain FDG uptake in C57Bl/6 mice (N = 37) plotted at the time of measurement. The dashed line is a smoothed spline curve through group averages. Both group averages (B) and within individual comparisons at different times of day (C) of total brain FDG uptake showed significantly higher uptake at the middle of the active phase at ZT 18. Error bars indicate SEM. * = Proc mixed, least square means contrast: P<0.005, P<0.001 and P<0.001 with ZT 6, ZT 12 and ZT 24/0. # = Paired t-test P<0.005.

Total FDG uptake in the brain differed significantly between different times of day (proc mixed, P<0.001). Figure 2a shows individual values for whole brain FDG uptake throughout the day. Whole brain FDG uptake centered around the mid of the dark phase of the LD cycle (ZT 18), are significantly increased, to 150% of the average of the other time points (Proc mixed, least square means contrast: P<0.005, P<0.001 and P<0.001 with ZT 6, ZT 12 and ZT 24/0 respectively, Figure 2b). The within individual comparisons are shown in Figure 2c and confirm that brain FDG uptake measurements, taken at ZT 18 and ZT 24/0 within the same mouse, differed significantly (paired t-test; P<0.005). No difference is seen for within-individual measurements of brain FDG uptake centered on ZT 6 and ZT 12 (paired t-test; P>0.05).

**Figure 3:**
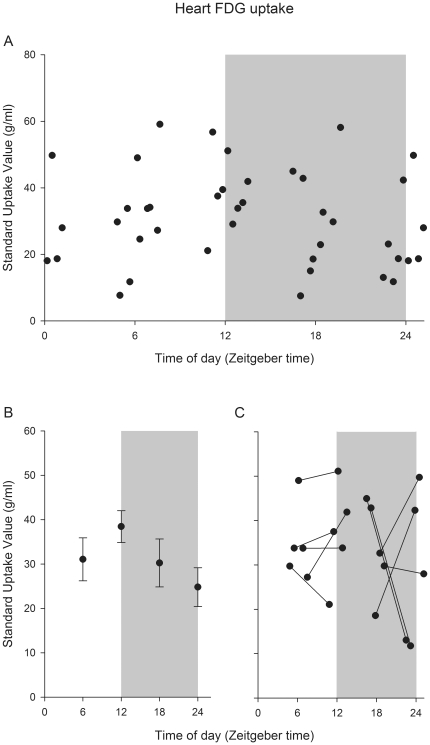
Time-of-day specific analysis of FDG uptake in heart. (A) Whole heart FDG uptake in the same C57Bl/6 mice as Figure 2 (N = 37) plotted at the time of measurement. There is no statistically significant variation of total FDG uptake for grouped averages (B). The within individual comparison (C) also shows no uniform FDG uptake at different times of the day.

In contrast to whole brain FDG uptake, heart FDG uptake shows no time-of-day difference (Figure 3a; proc mixed, P>0.05). Variability of heart FDG uptake was much larger than whole brain FDG uptake (compare Figure 2a to Figure 3a), and mean heart FDG uptake was similar for each of the four time points (Proc mixed, least square means contrast: P's>0.05; Figure 3b). Within individual comparison of heart FDG uptake confirmed no time-of-day variation between measurements centered on ZT 18 vs. ZT 24/0 and ZT 6 vs. ZT 12 (paired t-test, P's>0.05; Figure 3c).

**Figure 4:**
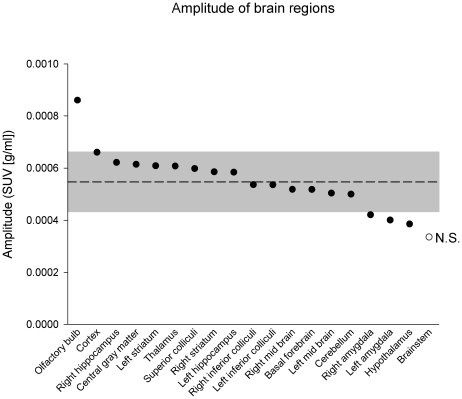
Amplitude of total FDG uptake in specific brain regions. All brain regions, except the brain stem, show significant variations with time (Proc mixed, α = 0.05). The dashed line indicates the average and gray area indicates average ± standard deviation.

Regional measurements of brain FDG uptake were established using a PET specific, mouse brain template. The regional mapping was validated through the correlation between a) the total FDG uptake as established by CT based volume-of-interest construction and b) the summation of the regional uptakes (Pearson R^2^ = 0.71; Figure S2). All brain regions, except the brain stem, showed significant variation in FDG uptake (average of voxel) over time (Proc mixed, α = 0.05; Figure 4). While all measured brain region, apart from the brain stem, showed peaking FDG uptake at ZT 18 (see Figure S3), the peak-to-trough amplitude of the nocturnal variation differs between the brain regions. Most tissues exhibited amplitudes in FDG uptake around the average (within one standard deviation), with some exceptions. The olfactory bulb and cortex show ‘above average’ amplitudes, while the amygdala and hypothalamus show ‘below average’ amplitudes. The brain stem showed the lowest amplitude, which confirms the absence of a statistically significant temporal variation in FDG uptake throughout the day. Total uptake (average uptake x volume (ccm)) for each of the brain regions is shown in Table 1.

**Table 1:** Total ^18^F-FDG uptake in brain regions.

Brain region	Total uptake(SUV [e^−05^ (g/ml)*ccm])
Cortex	23.42±0.87
Cerebellum	7.75±0.32
Brain stem	6.33±0.29
Olfactory bulb	4.29±0.18
Thalamus	4.05±0.17
Left striatum	2.06±0.07
Right striatum	2.01±0.07
Left hippocampus	1.85±0.08
Right hippocampus	1.72±0.07
Basal Forebrain	1.72±0.06
Superior colliculi	1.32±0.05
Hypothalamus	1.27±0.05
Right mid brain	1.20±0.05
Right amygdala	0.69±0.03
Central gray	0.61±0.03
Right inferior colliculi	0.56±0.02
Left amygdala	0.56±0.02
Left mid brain	0.33±0.01
Left inferior colliculi	0.22±0.01

## Discussion

Using positron emission tomography across the 24-hour day, we show that FDG uptake in the whole brain in living mice exhibits a nocturnal peak. Other studies have identified day-night or daily differences in 2-DG quantified glucose utilization in a relatively small number of brain regions, most with high glucose utilization during the dark phase of the LD cycle [9], [16]–[17]. Here, our *in vivo* data show strong 24-hour variation in FDG uptake within individual mice as determined by quantitative measurements in the brain. Although the brain structures are not always comparable due to different resolutions of the techniques (e.g. the ‘thalamus’ in our study is represented by 19 discrete nuclei in [17] of which only one is reported as showing a day-night difference, and 7 nuclei in [9] of which 4 show a difference), taken together these data indicate that while glucose uptake shows a 24-hour pattern in most brain regions, this pattern in uptake may not necessarily lead to a similar pattern in glucose utilization. For many rodent PET studies, the time-of-day of scanning and LD conditions are not strongly controlled variables, or not reported. One implication of our data is that not controlling for the time-of-day of scanning may result in a bias, within the range of physiologically relevant findings.

The within individual comparison is, to our knowledge, the first time brain glucose uptake in mice has been measured in such a time informed method. For the ZT 18 to ZT 24/0 comparison, all individuals show a decrease in FDG uptake. Strikingly, for the ZT 6 to ZT 12 comparison, all mice but one exhibit a drop in absolute brain FDG uptake. While this numerical drop in FDG uptake at the light to dark transition does not reach statistical significance, it appears rather consistent within the individual animal analysis, and may indicate that even minor aspects of the overall profile are still consistent. Animals were awake during FDG uptake and alive during PET scanning, and an unanaesthetized state has been shown to increase brain FDG uptake [25].

Differences are noted in the amplitudes of the 24-hour variation in FDG uptake in different brain regions. The SCN of the hypothalamus, containing the major circadian oscillator, is the only brain region reported in nocturnal rodents to show elevated glucose utilization as quantified using the 2-DG method during the light phase rather than during the night [9], [14], [17]. While the spatial resolution and ‘partial voluming effect’ [26] of our technique is not sufficient to identify brain regions on such a small scale, the below-average amplitude in the hypothalamus is indicative of an antiphase rhythm specifically in the SCN, that would quench the rhythmic profile expressed in the remaining hypothalamic nuclei. The low amplitude in both left and right amygdaloid FDG uptake may again be caused by rhythms in specific amygdaloid nuclei exhibiting different peak phases. This would be consistent with clock protein expression in rats being rhythmic in an antiphase relationship in the central nucleus of the amygdala and the basolateral amygdala [27].

Our regional analysis shows a comparatively large amplitude in average FDG uptake in the olfactory bulb, together with an overall average high uptake at all phases of the 24-hour cycle. Although a day-night variation in glucose utilization has been reported in the nucleus of the olfactory tract of the mouse [9], to our knowledge such a strong daily variation in glucose utilization of the olfactory bulb of the mouse has not been reported in previous studies in using the 2-DG method. The olfactory bulb can show *in vivo* and *in vitro* (SCN independent) rhythms in expression of the clock gene *period1*, as reported by a bioluminescent reporter in rats [28], [29]. This in contrast to other brain nuclei that show a larger dependency on signals derived from the SCN. Olfactory stimulation enhances light-induced responses in both transcriptional activity in the SCN and behavioral entrainment to a new LD cycle [30]. In our study, respiration anesthetics may have activated the olfactory bulb (in line with high overall uptake). Such activation would have been given at all times sampled and therefore did not introduce a 24-hour signal in itself. Indeed, it is feasible that this pattern is not visible in terminal experiments due to an absence of direct stimulation and the use of respiration anesthetics may have emphasized the intrinsic 24-hour pattern in the olfactory bulb not visible in other glucose uptake measurements.

Treatment of mice with 30 minutes of light of high intensity (only effects are shown for a reported intensity of a 1000 lux) induced *period* gene expression in the olfactory bulb itself [31]. There are SCN independent circadian rhythms in cedar oil induced c-FOS expression in the olfactory bulb and more interestingly, lesions of the mouse olfactory bulb shortened the period length of free running behavioral rhythms measured in DD, and a concordant enhancement of phase advances of the clock in the SCN, as inferred from behavioral rhythms [32]. Because of these data, it has been suggested that the olfactory bulb contains an independent, self-sustaining pacemaker. Our data highlights a strong 24-hour pattern of glucose uptake in the olfactory bulb and could support an important role for olfactory stimulation in the *in vivo* entrainment of the olfactory bulb clock specifically, as well as downstream entrainment of the SCN clock.

Heart rate changes can be rapid and heart rate is correlated to oxygen consumption and metabolic rate [33]. In response to an immediate increase in heart workload, glucose and lactate oxidation increases rapidly [34]. The absence of a 24-hour rhythm in FDG uptake is in contrast to the study by Karaganis *et al.* [18] on the diurnal chicken that revealed a nocturnal peak in glucose uptake in the heart as assessed by 2-DG autoradiography. Our data may simply reflect the rapid autonomic control of heart rate, which could be due in part to our paradigm, which subsequently affects FDG uptake (e.g. [35]). Balb/c mice show lower brain FDG uptake and higher heart FDG uptake when anesthetized with isoflurane during injection of the tracer and recovery [36]. Also in immunocompromised mice (C.B.-17 Scid/Scid strain), FDG uptake by the myocardium was increased by isoflurane anesthesia [37]. In our protocol, we have not kept the mice under isoflurane anesthesia during the interval between injection and scanning, and this may have benefited brain FDG uptake over heart FDG uptake. As a result, the signal to noise ratio in heart FDG uptake may have increased, resulting in large inter-individual variability and lower likelihood of finding time-of-day specific variability. Also, in humans, plasma glucose levels have been shown to affect myocardial FDG uptake. Fasting before imaging decrease plasma FDG clearance rates and decreases myocardial FDG uptake [38], but can also result in larger variability [39], [40].

The brain cannot store large amounts of glucose, and is largely dependent on blood glucose levels to supply energy sources. In rodents, plasma glucose levels show circadian variation, peaking around the time of early activity in the dark phase in the rest-activity cycle, which is independent of feeding rhythms [41]–[43]. The rhythm in glucose availability shows strong dependence on the SCN (see [44]), and also the peripheral clock in the liver has been shown to be involved in glucose homeostasis [45]. While skeletal muscle is commonly accepted as the largest glucose utilizing tissue, the brain is also a large glucose metabolizing tissue. The brain is often reported to use a large portion of the available glucose, in the range of 25% of total body glucose utilization in humans [46], [47], and is responsible for approximately 20% and 3% (in humans and rats, respectively) of resting metabolic state [20]. Our data represent a measure of glucose uptake, and caution is warranted in extrapolating these measures to brain glucose utilization. The similarities in the peak timing of brain glucose uptake and blood glucose availability is intuitive, but may also indicate a potential vulnerability of the brain to alterations in the circadian profile of blood glucose concentration. It may be functionally related that restricting food to certain times of the day only affects the food entrainable oscillator and not the SCN, while caloric restriction alters SCN entrainment (for review see [48]).

What are the functional correlates of the daily variation in glucose uptake in the brain? Indeed we know that central self-sustained clocks (e.g. the master pacemaker located in the SCN of the hypothalamus), and driven oscillations in clock gene expression can regulate many rhythmic processes in biochemistry, physiology and behavior allowing anticipation of environmental changes [6], [7]. Sustaining these anticipatory rhythms and the processes they drive undoubtedly requires energy. Electrical activity of the brain shows wake-dependent and circadian modulation in humans [49], and there is substantial evidence that cognitive performance is subject to circadian modulation [50]. Also, our 24-hour variation in vigilance will affect brain activity and metabolism. The daily variation in brain metabolic activity is made up of two groups of processes that can vary with the time of day; diurnal variation in processes driven by the brain itself, and processes that are driven by other aspects of physiology that demand brain activity.

In summary, our data show strong *in vivo* 24-hour patterns in overall brain glucose uptake, peaking in the mid-dark phase, which is the active phase for the nocturnal mouse. Daily patterning is apparent in almost all brain regions, but there is heterogeneity in terms of amplitude, possibly resulting from sub-brain region dynamics. These data, first of all, underline the importance of controlling for the time-of-day of PET scanning during studies of the CNS. A practical implication for clinical oncology of a daily rhythm in brain FDG uptake could be a decrease of ‘biological variation’ through standardization, or temporal waveform normalization of FDG measurements. Previous studies using the 2-DG method have shown that a relatively small number of brain structures exhibiting a day-night difference in glucose utilization, whilst our data show a daily 24 hour pattern of underlying glucose uptake in almost all brain regions identified. Our data describe a significant, high amplitude daily rhythm in glucose uptake throughout the brain, which is reproducibly observed within individual animals.

## Materials and Methods

### Animals and experimental protocol

Mice were kept in light-tight cabinets which were climate controlled (19–21°C, 60–70% humidity), and food and water were available *ad libitum*. Male (N = 6) and female (N = 13) adult C57Bl/6 mice were bred at the University of Notre Dame and housed with littermates, and mice were randomly assigned to one of four groups (N = 4–5 per group). Each group was entrained to a 12 hour light∶12 hour dark (12∶12 LD) cycle for at least 3 weeks, where LD cycles for two groups were 12 hours out of phase with the LD cycles of the other two groups (lights on at 03:00 AM and 15:00 PM respectively). On the day before measurement, mice were individually housed and food was removed at least 5 hours before the time of scanning. Longitudinal dual-modality CT and PET scanning has been employed for over 10 years [51] and has minimal effect on mouse health under our experimental conditions, and all experiments were approved by the University of Notre Dame Animal Care and Use Committee. Experiments were performed in accordance with NIH Guidelines for the Care and Use of Laboratory Animals.

**Schedule 1** was aimed at measuring FDG uptake over a 24-hour period, centered around 4 time points (lights on, mid-light, lights off and mid-dark). Animals were 64±3 (SEM) days of age at the time of measurement. Two groups of mice that were entrained to LD cycles that were 12 hours out of phase were measured on the morning of one day, and the other two groups were measured on the afternoon of a second day. On both days, mice from the two anti-phase groups were measured in alternating order, 20 minutes apart. Measurement times were chosen such that group mean measuring times were Zeitgeber Time (ZT [time of lights on is ZT 0]) 06:15 (±00:59 hours), ZT 12:10 (±00:54), ZT 18:08 (±01:04) and ZT 23:50 (±00:54) (see Figure S1a). These groups will be referred to as ZT 6, ZT 12, ZT 18 and ZT 24/0 respectively.

**Schedule 2** was aimed at testing reproducibility between, and within individuals. The 2 groups of mice that were measured at ZT 6 and ZT 18, we re-mixed into two groups and re-entrained to the LD cycles. Measurements were made centered around light and dark, and 10 days later, in the same mice, centered on mid-light and mid-dark (see Figure S1b).

### PET data acquisition and analysis

Mice were measured with 20 minute intervals, alternating mice between two groups entrained to LD cycles 12 hours apart. Mice were anesthetized with 1.5% isoflurane in an induction box, and retro-orbitally [52], [53] injected with ∼200 µCI (Mean = 201.4, SEM = 1.4) of (^18^F) fluorodeoxyglucose (FDG [Spectron MRC, South Bend, IN, USA]). Mice that were injected in the dark phase of the LD cycle were briefly exposed to ≤5 minutes of light during the injection procedure and were subsequently placed back into the dark. The clock only fully resets after 2 hours but not start before 1 hour after the initiation of the light pulse [54], and the light is thus unlikely to have influenced the subsequent 30–45 minutes of FDG uptake and PET scanning. After injection, exactly 30 minutes were allowed for FDG uptake, before scanning started. Within one minute after FDG injection mice recovered from anesthesia, and mice did not receive anesthesia during the FDG uptake period. Prior to scanning, mice were again anesthetized with isoflurane and placed inside the scanner, secured in a tooth bar. X-ray computed tomography (CT) and Positron emission tomography (PET) images were acquired in an Albira scanner (Carestream Health, Rochester, NY, USA). High density PET (voxel size 0.65×0.65×0.944 mm [xyz]) and CT (voxel size 250 Hounsfield units) images were reconstructed and regional FDG uptake was quantified in PMOD version 3.2 (PMOD technologies, Zurich, Switzerland).

For the brain, the volume-of-interest was determined using the scull outline of individual mice in the CT images and FDG uptake was quantified from the reconstructed, and fused PET images. Measures of localized FDG uptake in pre-defined brain regions was obtained through applying a mouse brain template directly to the PET image. In short, the acquired PET image of the mouse was masked such that only the head portion was available and the image was manually co-registered with the general PET mouse brain mask available in the PMOD program. The PET image was then normalized to the mask using PMOD 9.2 normalization algorithms, and overlaid with the mouse brain template [55], [56]. Individual amplitude of regional FDG uptake was expressed as the difference between the lowest and highest mean voxel values. The amplitudes were categorized in three groups, an above average, an intermediate group that encompasses amplitudes with the range of the mean ±1 standard deviation, and a below average group. Heart FDG uptake was determined from PET images using the region-of-interest assessed from the PET directly.

FDG uptake was expressed as Standard Uptake Values (SUV [g/ml]), accounting for body mass and minor variations in injected dose. For assessing time-of-day differences, data of schedule 1 and 2 were combined. Because of the non-equidistant nature of the sampling, data was binned in four groups centered on ZT 6, ZT12, ZT 18 and ZT 24/0.

### Statistics

Time-of-day effects were tested by fitting mixed linear models, accounting for repeated measurements when applicable (proc mixed, SAS version 9.2 [SAS Institute, Cary, NC, USA]) with post-hoc least square means (LSMEANS) contrast analysis. Within individual differences in schedule 2 were tested using paired t-tests (proc t-test, SAS version 9.2). For all statistical analysis a cutoff of p<0.05 was applied.

## Supporting Information

Figure S1 **Graphical representation of the layout of the experiment, indicating two schedules.** Schedule 1 was aimed to measure FDG uptake, centered around 4 time points. Schedule 2 was aimed at testing reproducibility between, and within individuals.
(TIF)

Figure S2 **Correlation between total FDG uptake established through CT-based brain region-of-interest and summation of all regional brain FDG uptake values.** The line indicates the linear regression. The spearman rank correlation (R^2^ = 0.71) indicates the correlation between the two methods.
(TIF)

Figure S3 **FDG uptake in specific brain regions.** All brain regions, except the brain stem, show significant variations with time. Graphs are sorted left to right, top to bottom, in order of above average to below average amplitude of the rhythm. P values indicate the significance value for time-of-day specific effects.
(TIF)

## References

[1] Hjelstuen OK, Svadberg A, Olberg DE, Rosser M (2011). Standardization of fluorine-18 manufacturing processes: new scientific challenges for PET.. Eur J Pharm Biopharm.

[2] Pauwels EK, Ribeiro MJ, Stoot JH, McCready VR, Bourguignon M (1998). FDG accumulation and tumor biology.. Nucl Med Biol.

[3] Basu S, Alavi A (2008). Unparalleled contribution of 18F-FDG PET to medicine over 3 decades.. J Nucl Med.

[4] Schnockel U, Hermann S, Stegger L, Law M, Kuhlmann M (2010). Small-animal PET: a promising, non-invasive tool in pre-clinical research.. Eur J Pharm Biopharm.

[5] Boellaard R, O'Doherty MJ, Weber WA, Mottaghy FM, Lonsdale MN (2011). FDG PET and PET/CT: EANM procedure guidelines for tumour PET imaging: version 1.0.. Eur J Nucl Med Mol Imaging.

[6] Welsh DK, Takahashi JS, Kay SA (2010). Suprachiasmatic nucleus: cell autonomy and network properties.. Annu Rev Physiol.

[7] Klein DC, Moore RY, Reppert SM (1991). Suprachiasmatic Nucleus: The Mind's Clock.

[8] Sokoloff L, Reivich M, Kennedy C, Des Rosiers MH, Patlak CS (1977). The [14C]deoxyglucose method for the measurement of local cerebral glucose utilization: theory, procedure, and normal values in the conscious and anesthetized albino rat.. J Neurochem.

[9] Jay TM, Jouvet M, des Rosiers MH (1985). Local cerebral glucose utilization in the free moving mouse: a comparison during two stages of the activity-rest cycle.. Brain Res.

[10] Rivkees SA, Fox CA, Jacobson CD, Reppert SM (1988). Anatomic and functional development of the suprachiasmatic nuclei in the gray short-tailed opossum.. J Neurosci.

[11] Schwartz WJ, Reppert SM, Eagan SM, Moore-Ede MC (1983). *In vivo* metabolic activity of the suprachiasmatic nuclei: a comparative study.. Brain Res.

[12] Cassone VM (1988). Circadian variation of [14C]2-deoxyglucose uptake within the suprachiasmatic nucleus of the house sparrow, *Passer domesticus*.. Brain Res.

[13] Cantwell EL, Cassone VM (2002). Daily and circadian fluctuation in 2-deoxy[(14)C]-glucose uptake in circadian and visual system structures of the chick brain: effects of exogenous melatonin.. Brain Res Bull.

[14] Schwartz WJ, Klein DC, Moore RY, Reppert SM (1991). SCN metabolic activity *in vivo*.. Suprachiasmatic Nucleus: The Mind's Clock.

[15] Guilding C, Piggins HD (2007). Challenging the omnipotence of the suprachiasmatic timekeeper: are circadian oscillators present throughout the mammalian brain?. Eur J Neurosci.

[16] Crane PD, Braun LD, Cornford EM, Nyerges AM, Oldendorf WH (1980). Cerebral cortical glucose utilization in the conscious rat: evidence for a circadian rhythm.. J Neurochem.

[17] Room P, Tielemans AJ (1989). Circadian variations in local cerebral glucose utilization in freely moving rats.. Brain Res.

[18] Karaganis SP, Bartell PA, Shende VR, Moore AF, Cassone VM (2009). Modulation of metabolic and clock gene mRNA rhythms by pineal and retinal circadian oscillators.. Gen Comp Endocrinol.

[19] Attwell D, Laughlin SB (2001). An energy budget for signaling in the grey matter of the brain.. J Cereb Blood Flow Metab.

[20] Rolfe DF, Brown GC (1997). Cellular energy utilization and molecular origin of standard metabolic rate in mammals.. Physiol Rev.

[21] Willich SN (1999). Circadian variation and triggering of cardiovascular events.. Vasc Med.

[22] Davidson AJ, London B, Block GD, Menaker M (2005). Cardiovascular tissues contain independent circadian clocks.. Clin Exp Hypertens.

[23] Durgan DJ, Hotze MA, Tomlin TM, Egbejimi O, Graveleau C (2005). The intrinsic circadian clock within the cardiomyocyte.. Am J Physiol Heart Circ Physiol.

[24] Young ME, Razeghi P, Cedars AM, Guthrie PH, Taegtmeyer H (2001). Intrinsic diurnal variations in cardiac metabolism and contractile function.. Circ Res.

[25] Shimoji K, Ravasi L, Schmidt K, Soto-Montenegro ML, Esaki T (2004). Measurement of cerebral glucose metabolic rates in the anesthetized rat by dynamic scanning with 18F-FDG, the ATLAS small animal PET scanner, and arterial blood sampling.. J Nucl Med.

[26] Soret M, Bacharach SL, Buvat I (2007). Partial-volume effect in PET tumor imaging.. J Nucl Med.

[27] Lamont EW, Robinson B, Stewart J, Amir S (2005). The central and basolateral nuclei of the amygdala exhibit opposite diurnal rhythms of expression of the clock protein Period2.. Proc Natl Acad Sci U S A.

[28] Abe M, Herzog ED, Yamazaki S, Straume M, Tei H (2002). Circadian rhythms in isolated brain regions.. J Neurosci.

[29] Abraham U, Prior JL, Granados-Fuentes D, Piwnica-Worms DR, Herzog ED (2005). Independent circadian oscillations of *Period1* in specific brain areas *in vivo* and *in vitro*.. J Neurosci.

[30] Amir S, Cain S, Sullivan J, Robinson B, Stewart J (1999). Olfactory stimulation enhances light-induced phase shifts in free-running activity rhythms and Fos expression in the suprachiasmatic nucleus.. Neuroscience.

[31] Hamada T, Honma S, Honma K (2011). Light responsiveness of clock genes, *Per1* and *Per2*, in the olfactory bulb of mice.. Biochem Biophys Res Commun.

[32] Granados-Fuentes D, Tseng A, Herzog ED (2006). A circadian clock in the olfactory bulb controls olfactory responsivity.. J Neurosci.

[33] Green JA (2011). The heart rate method for estimating metabolic rate: review and recommendations.. Comp Biochem Physiol A Mol Integr Physiol.

[34] Goodwin GW, Taylor CS, Taegtmeyer H (1998). Regulation of energy metabolism of the heart during acute increase in heart work.. J Biol Chem.

[35] Taegtmeyer H (2011). Tracing cardiac metabolism *in vivo*: one substrate at a time.. J Nucl Med.

[36] Toyama H, Ichise M, Liow JS, Vines DC, Seneca NM (2004). Evaluation of anesthesia effects on [18F]FDG uptake in mouse brain and heart using small animal PET.. Nucl Med Biol.

[37] Fueger BJ, Czernin J, Hildebrandt I, Tran C, Halpern BS (2006). Impact of animal handling on the results of 18F-FDG PET studies in mice.. J Nucl Med.

[38] Kreissl MC, Stout DB, Wong KP, Wu HM, Caglayan E (2011). Influence of dietary state and insulin on myocardial, skeletal muscle and brain [F]-fluorodeoxyglucose kinetics in mice.. EJNMMI Res.

[39] Gropler RJ, Siegel BA, Lee KJ, Moerlein SM, Perry DJ (1990). Nonuniformity in myocardial accumulation of fluorine-18-fluorodeoxyglucose in normal fasted humans.. J Nucl Med.

[40] Knuuti MJ, Nuutila P, Ruotsalainen U, Saraste M, Harkonen R (1992). Euglycemic hyperinsulinemic clamp and oral glucose load in stimulating myocardial glucose utilization during positron emission tomography.. J Nucl Med.

[41] Pauly JE, Scheving LE (1967). Circadian rhythms in blood glucose and the effect of different lighting schedules, hypophysectomy, adrenal medullectomy and starvation.. Am J Anat.

[42] La Fleur SE, Kalsbeek A, Wortel J, Buijs RM (1999). A suprachiasmatic nucleus generated rhythm in basal glucose concentrations.. J Neuroendocrinol.

[43] Cailotto C, La Fleur SE, Van Heijningen C, Wortel J, Kalsbeek A (2005). The suprachiasmatic nucleus controls the daily variation of plasma glucose via the autonomic output to the liver: are the clock genes involved?. Eur J Neurosci.

[44] Kalsbeek A, Scheer FA, Perreau-Lenz S, La Fleur SE, Yi CX (2011). Circadian disruption and SCN control of energy metabolism.. FEBS Lett.

[45] Lamia KA, Storch KF, Weitz CJ (2008). Physiological significance of a peripheral tissue circadian clock.. Proc Natl Acad Sci U S A.

[46] Meyer C, Dostou JM, Welle SL, Gerich JE (2002). Role of human liver, kidney, and skeletal muscle in postprandial glucose homeostasis.. Am J Physiol Endocrinol Metab.

[47] DeFronzo RA (2004). Pathogenesis of type 2 diabetes mellitus.. Med Clin North Am.

[48] Challet E (2010). Interactions between light, mealtime and calorie restriction to control daily timing in mammals.. J Comp Physiol B.

[49] Aeschbach D, Matthews JR, Postolache TT, Jackson MA, Giesen HA (1997). Dynamics of the human EEG during prolonged wakefulness: evidence for frequency-specific circadian and homeostatic influences.. Neurosci Lett.

[50] Schmidt C, Collette F, Cajochen C, Peigneux P (2007). A time to think: circadian rhythms in human cognition.. Cogn Neuropsychol.

[51] Beyer T, Townsend DW, Brun T, Kinahan PE, Charron M (2000). A combined PET/CT scanner for clinical oncology.. J Nucl Med.

[52] Nanni C, Pettinato C, Ambrosini V, Spinelli A, Trespidi S (2007). Retro-orbital injection is an effective route for radiopharmaceutical administration in mice during small-animal PET studies.. Nucl Med Commun.

[53] Kim C, Kim IH, Kim S, Kim YS, Kang SE (2011). Comparison of the intraperitoneal, retroorbital and per oral routes for F-18 FDG administration as effective alternatives to intravenous administration in mouse tumor models using small animal PET/CT Studies.. Nucl Med Mol Imaging.

[54] Best JD, Maywood ES, Smith KL, Hastings MH (1999). Rapid resetting of the mammalian circadian clock.. J Neurosci.

[55] Ma Y, Hof PR, Grant SC, Blackband SJ, Bennett R (2005). A three-dimensional digital atlas database of the adult C57BL/6J mouse brain by magnetic resonance microscopy.. Neuroscience.

[56] Mirrione MM, Schiffer WK, Fowler JS, Alexoff DL, Dewey SL (2007). A novel approach for imaging brain-behavior relationships in mice reveals unexpected metabolic patterns during seizures in the absence of tissue plasminogen activator.. Neuroimage.

