# Supplementary figures and images for: A 24-Hour Temporal Profile of In Vivo Brain and Heart PET Imaging Reveals a Nocturnal Peak in Brain 18F-Fluorodeoxyglucose Uptake
Source: PLoS One. 2012 Feb 22;7(2):e31792. doi: 10.1371/journal.pone.0031792 (PMC3285174; doi:10.1371/journal.pone.0031792)

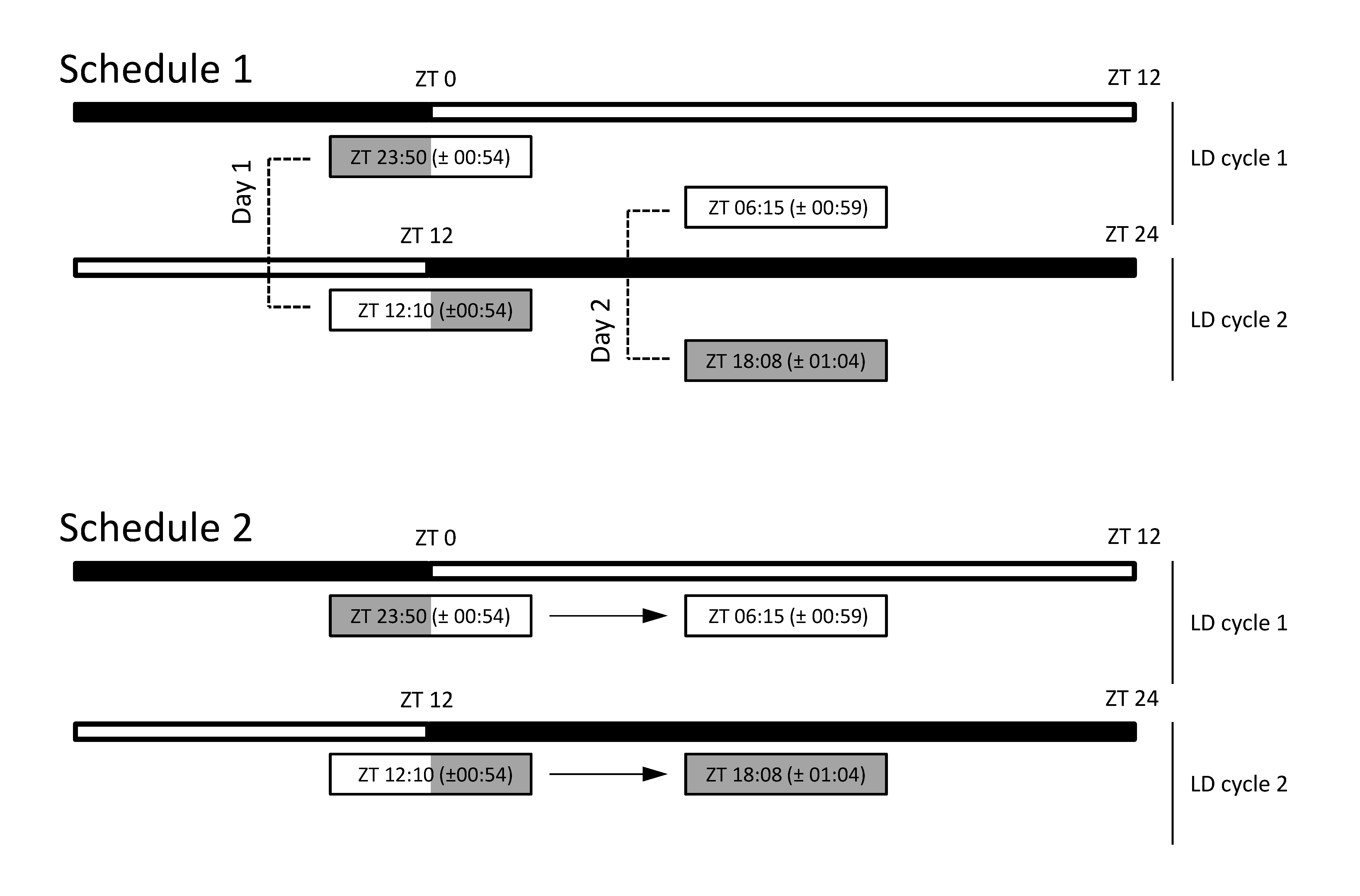

Supplement: Figure S1 — Graphical representation of the layout of the experiment, indicating two schedules. Schedule 1 was aimed to measure FDG uptake, centered around 4 time points. Schedule 2 was aimed at testing reproducibility between, and within individuals. (TIF) [file pone.0031792.s001.tif]

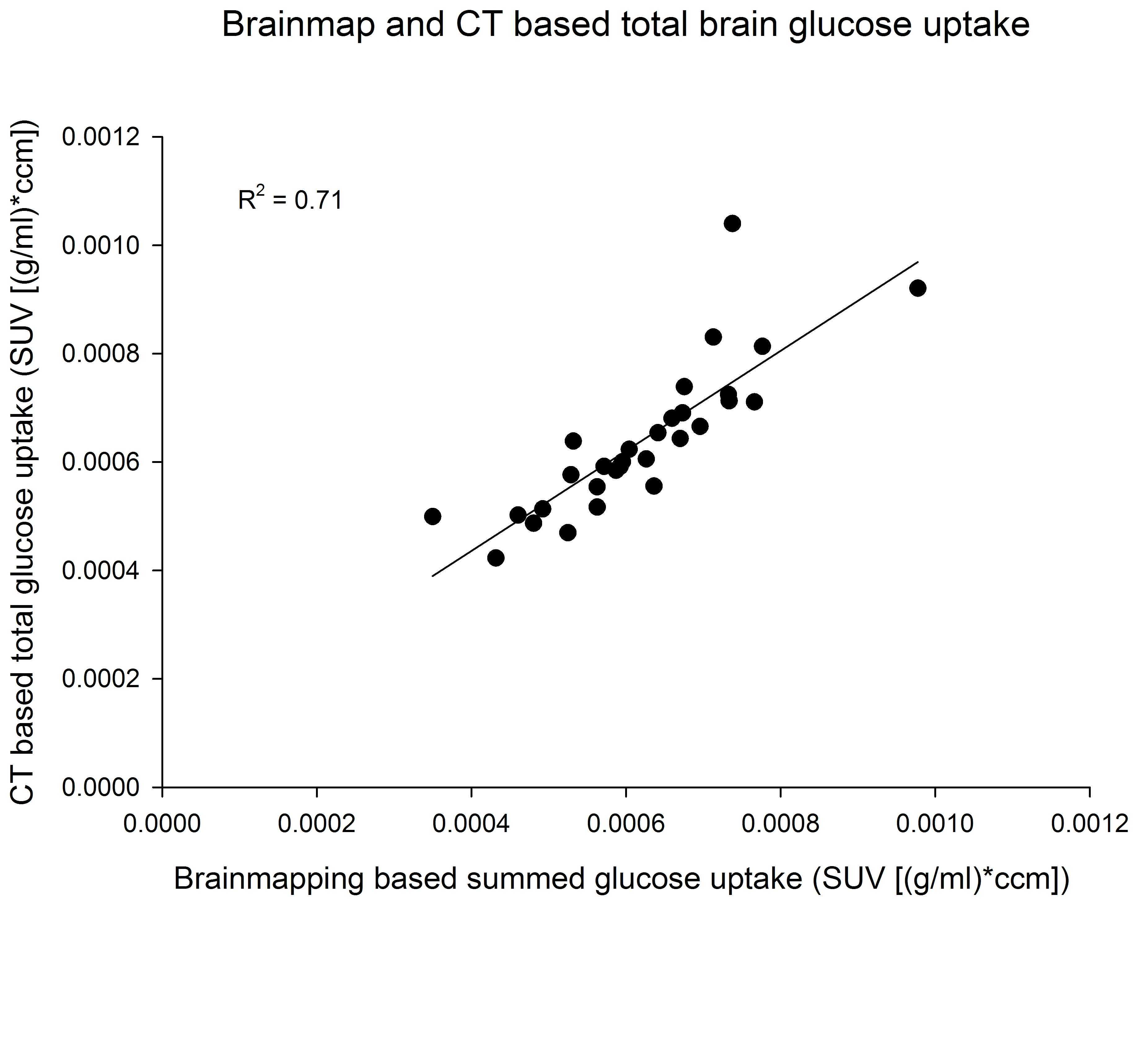

Supplement: Figure S2 — Correlation between total FDG uptake established through CT-based brain region-of-interest and summation of all regional brain FDG uptake values. The line indicates the linear regression. The spearman rank correlation (R2 = 0.71) indicates the correlation between the two methods. (TIF) [file pone.0031792.s002.tif]

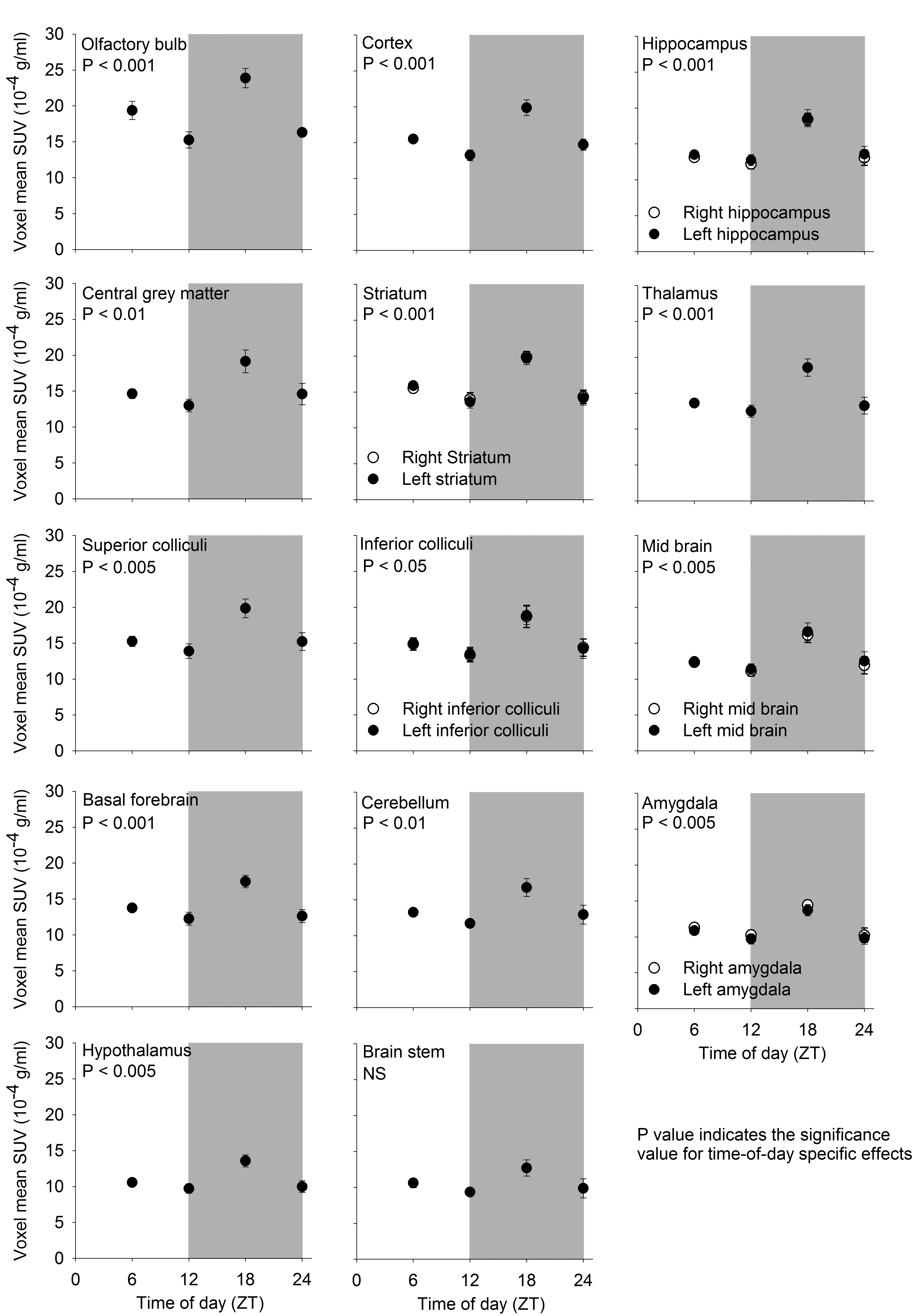

Supplement: Figure S3 — FDG uptake in specific brain regions. All brain regions, except the brain stem, show significant variations with time. Graphs are sorted left to right, top to bottom, in order of above average to below average amplitude of the rhythm. P values indicate the significance value for time-of-day specific effects. (TIF) [file pone.0031792.s003.tif]
